# Recurrent Reactive Infectious Mucocutaneous Eruption (RIME) in a Pediatric Patient Triggered by Mycoplasma pneumoniae and Enterovirus Coinfection

**DOI:** 10.7759/cureus.93698

**Published:** 2025-10-02

**Authors:** Mohamad B Alebaji, Anna Tryfonos, Bhavya Vasantham, Ratna Basak

**Affiliations:** 1 Pediatrics, State University of New York Downstate Health Sciences University, New York, USA; 2 Pediatrics, NYC Health + Hospitals/Kings County, New York, USA

**Keywords:** conjunctival injection, general pediatrics, mucocutaneous eruption, mucosal lesions, mycoplasma pneumoniae, oral ulcers, pediatric dermatology, reactive infectious mucocutaneous eruption, rime, viral exanthem

## Abstract

Reactive infectious mucocutaneous eruption (RIME) is an underrecognized condition often misdiagnosed as Stevens-Johnson syndrome (SJS). We present a case of a 10-year-old boy who presented with fever, cough, painful oral ulcers, and conjunctival injection that progressed to severe mucositis with urethral involvement. This case is notable both for the unusual urethral manifestation and for its association with a rare co-infection by *Mycoplasma pneumoniae* and enterovirus, attributable to RIME.

## Introduction

Reactive infectious mucocutaneous eruption (RIME) represents a spectrum of post-infectious mucocutaneous diseases that primarily affect children and adolescents [1]. It is characterized by severe mucositis, which may occur with or without accompanying skin lesions. Formerly known as *Mycoplasma*-induced rash and mucositis (MIRM), the condition is now referred to as RIME to reflect its association with a broader range of pathogens, including *Chlamydia pneumoniae*, enteroviruses, and other respiratory pathogens [1, 2].

Among the various infectious triggers, *Mycoplasma pneumoniae *remains the most common cause of pediatric RIME. As a leading pathogen responsible for community-acquired pneumonia in children, it has a well-established link between respiratory infection and mucocutaneous manifestations [3, 4, 5].

Although RIME generally follows a self-limiting course, its diagnosis and management remain challenging, particularly in pediatric patients, due to its incompletely understood pathophysiology and variable immune response [5]. While recurrent RIME is considered rare, it has been reported in association with sequential infections and, in very limited cases, with co-infections involving multiple pathogens [6]. The exact role of coinfection in amplifying mucocutaneous manifestations remains unclear and is still under investigation. Given the limited documentation of recurrent RIME in children, our report is distinctive in describing both urethral involvement and a dual infection with *Mycoplasma pneumoniae *and enterovirus.

## Case presentation

**Figure 1:**
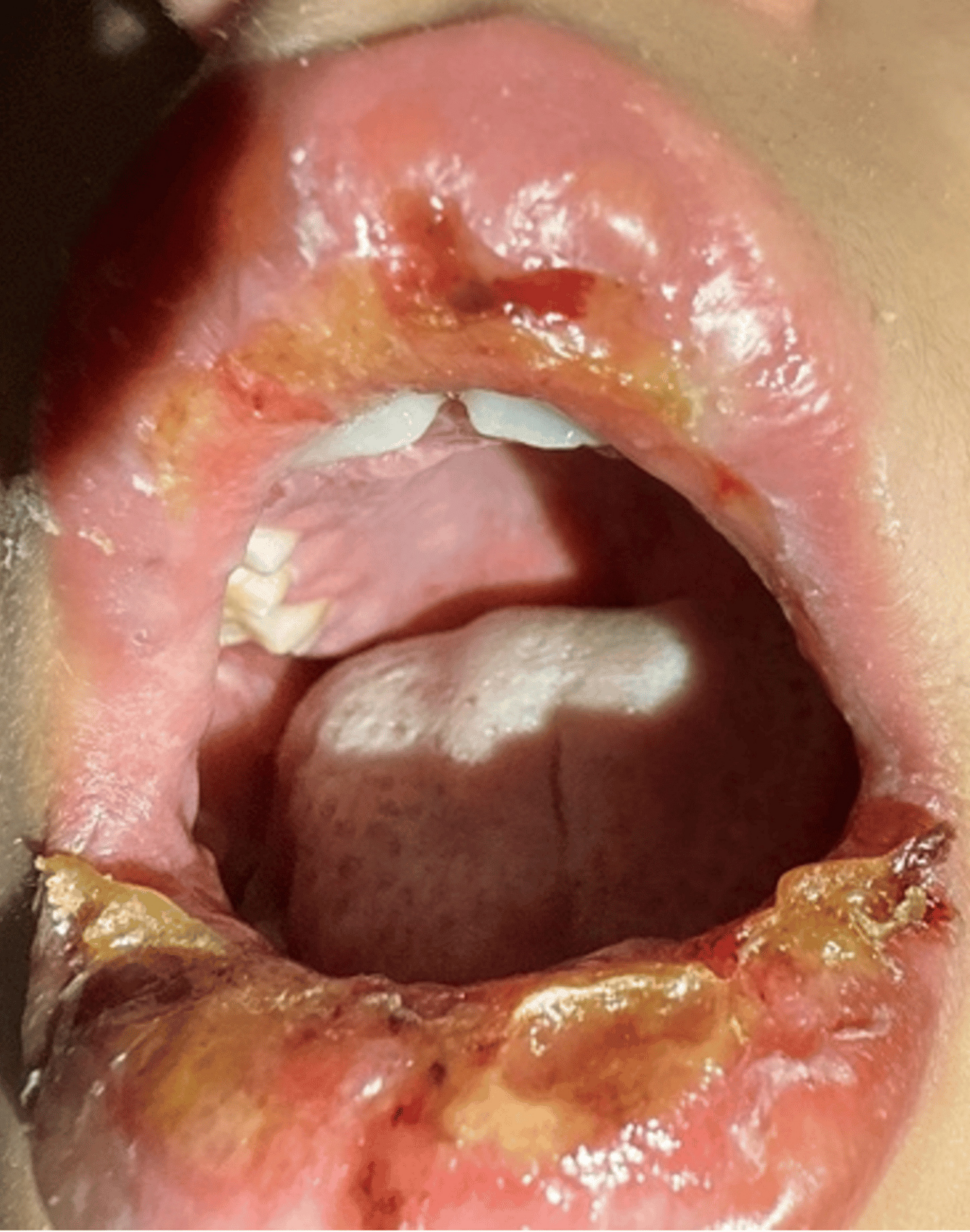
Mouth lesions, significant erythema, ulceration, and yellow crusting present on the swollen upper and lower lips with involvement of the oral mucosa, including sloughing and extension to the buccal mucosa.

**Figure 2:**
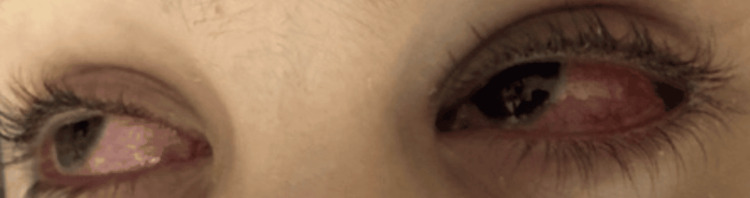
Bilateral conjunctival injection, erythema, chemosis, and mild eyelid swelling, with no apparent discharge

A 10-year-old boy presented to the emergency department with a one-week history of fever, cough, and decreased oral intake. Over the preceding three days, he had developed painful oral ulcers, crusted lips (Figure 1), and conjunctival injection with tearing in both eyes (Figure 2).

**Figure 3:**
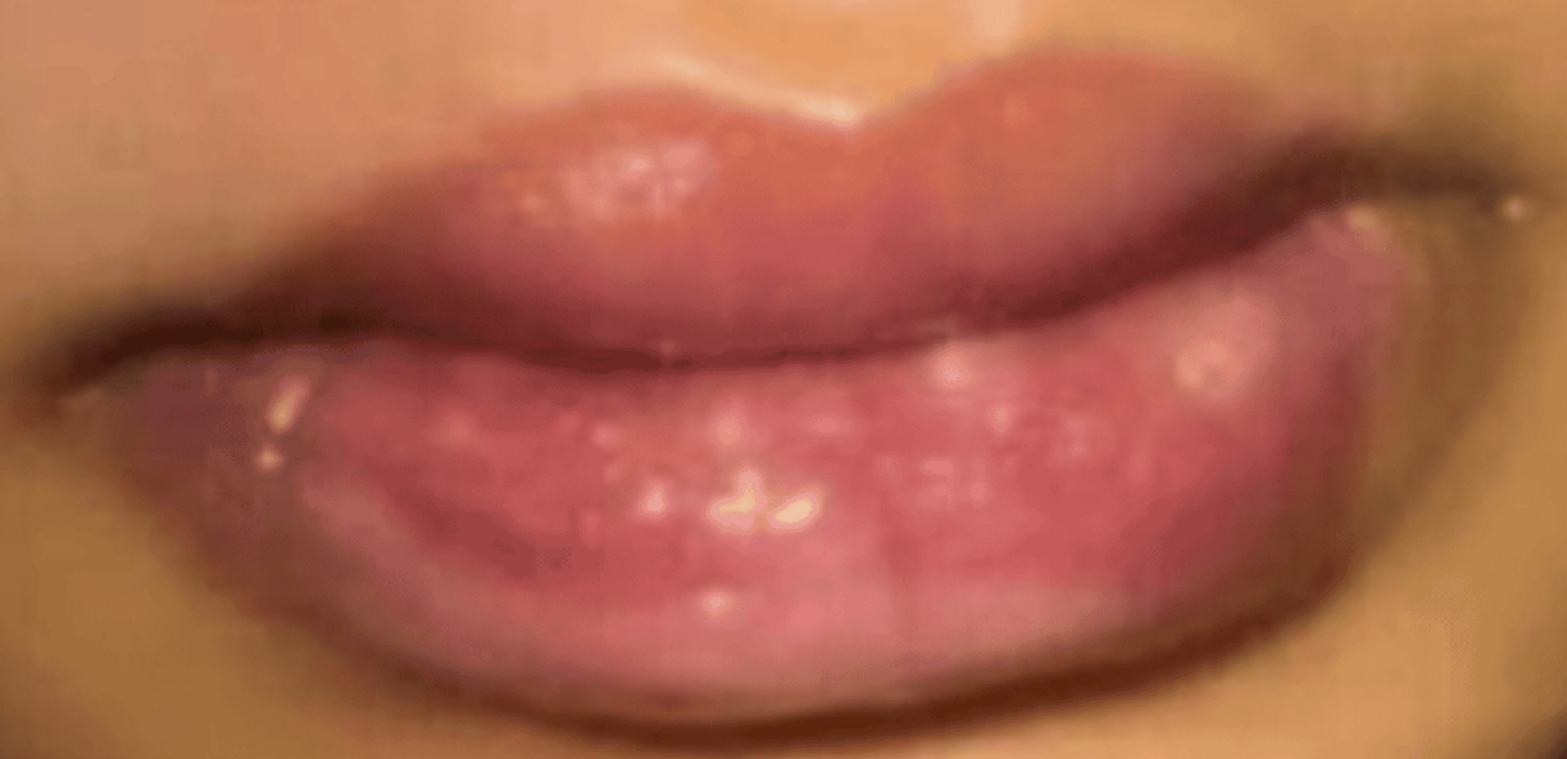
Three days prior to presentation, showing milder symptoms with mildly swollen and crusted lips and early mucosal involvement. No significant ulceration or bleeding is evident at this stage.

Two days before this visit, he had been evaluated in the ED for similar but milder oral lesions (Figure 3).

At that time, his symptoms were mild, and he was discharged with supportive care. His mother noted that one year prior, he had experienced similar self-limiting symptoms and medical records showed that he had tested positive for Influenza A that time.

**Figure 4:**
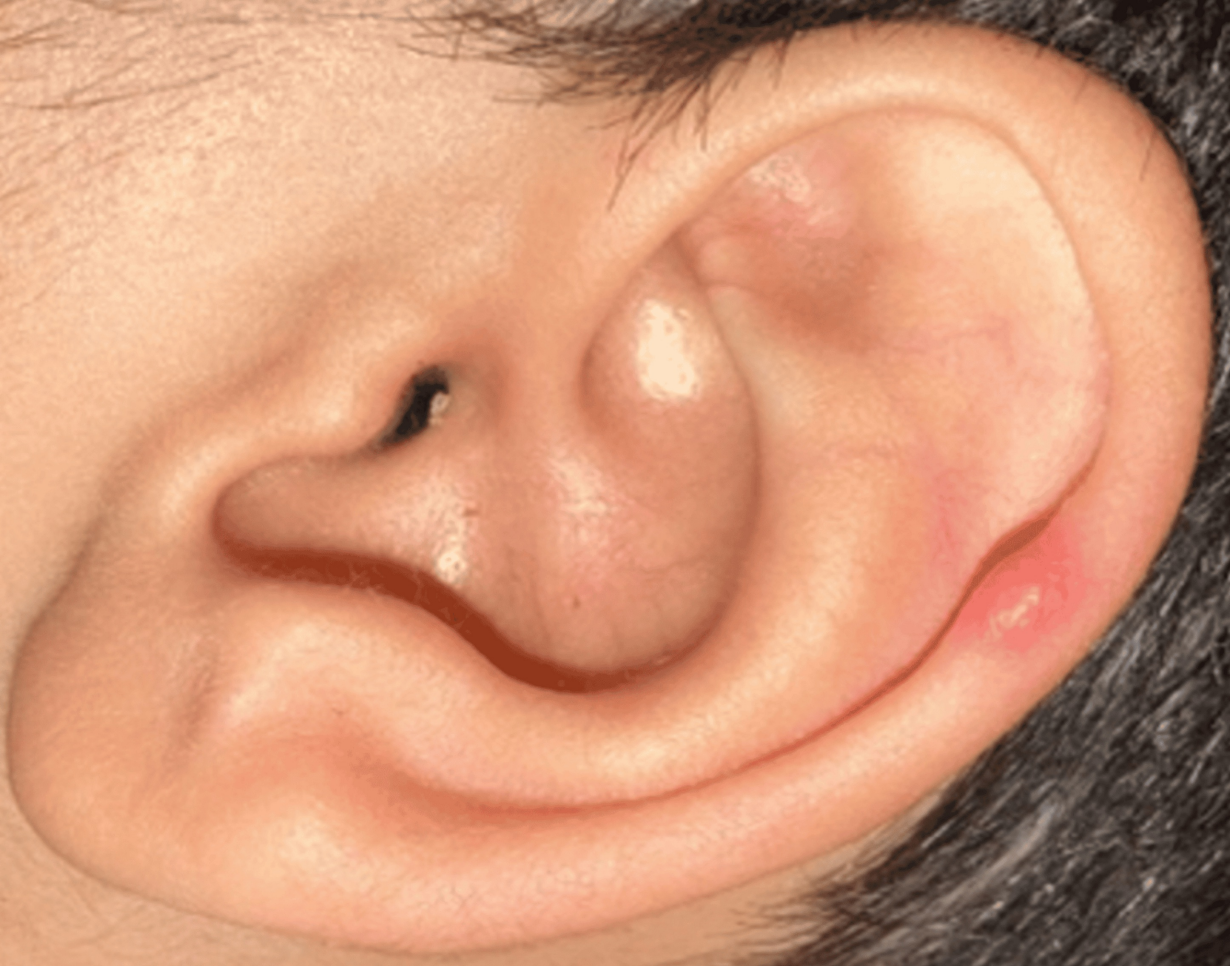
Scattered pink papules observed on the ear lobe. Similar lesions were noted on the nasal ala.

During the current presentation, the patient exhibited extensive buccal mucosal erosions, conjunctival injection, and scattered pink papules on the nasal ala and ear lobes (Figure 4).

On systemic examination, he was afebrile with stable vital signs, no lymphadenopathy, and no hepatosplenomegaly. Other skin areas were carefully examined and found to be clear, with no targetoid or widespread lesions, thereby helping to distinguish from Stevens-Johnson Syndrome (SJS) or erythema multiforme (EM).

Initial laboratory tests were notable for a mildly elevated C-reactive protein and positive results for rhinovirus/enterovirus and *Mycoplasma* infection (Table 1).

**Table 1:** Summary of initial laboratory investigations

Test	Result	Reference Range
White Blood Cell Count (WBC)	10.64 × 10⁹/L	4.5–13.5 × 10⁹/L
C-Reactive Protein (CRP)	30 mg/L	0.1–2.80 mg/L
Procalcitonin (PCT)	0.05 ng/mL	<0.5 ng/mL
Erythrocyte Sedimentation Rate (ESR)	27 mm/hr	0–15 mm/hr
Liver Function Tests	Normal	Normal
GenMark Respiratory Panel	Positive for Rhinovirus/Enterovirus	-
Herpes simplex virus (HSV) PCR	Negative	-
Mycoplasma IgM	Positive (3.91)	<0.9 (Negative)
Mycoplasma PCR	Positive	-
Alanine Aminotransferase (ALT)	13 U/L	7–56 U/L
Aspartate Aminotransferase (AST)	33 U/L	10–40 U/L
Total Bilirubin	0.5 mg/dL	0.1–1.2 mg/dL

The ED images from his previous visit (Figure 3) demonstrated progression of his lesions within the last 48 hours. He was unable to take in oral fluids; hence, he was admitted for dehydration management.

During hospitalization, he reported mild dysuria, and examination revealed a new lesion at the tip of his urethral meatus that had not been noted at admission. A urinalysis was unremarkable, and the urology team was consulted, recommending observation only.

The combination of mucosal erosions, conjunctival injection, and oral ulcers prompted consideration of Stevens-Johnson Syndrome (SJS), herpetic gingivostomatitis, and Kawasaki disease. SJS was ruled out due to the absence of systemic toxicity, minimal skin involvement, and no recent medication use. Herpetic gingivostomatitis was excluded based on a negative herpes simplex virus PCR test and the absence of vesicular lesions. Kawasaki disease was considered unlikely given the short history of fever and no extremity changes.

The patient was treated with azithromycin (5 mg/kg/day once daily) and supportive measures, which included analgesics and oral hydration. By the fifth day of hospitalization, the oral lesions showed significant improvement, and he was discharged with follow-up instructions. At a clinic visit one week after discharge, he appeared stable with no new complaints or recurrence of symptoms. The clinical picture, positive for *Mycoplasma pneumoniae *and rhinovirus infection, along with his response to azithromycin, supported a diagnosis of reactive infectious mucocutaneous eruption (RIME). The favorable response to treatment further reinforced this diagnosis.

## Discussion

Reactive infectious mucocutaneous eruption (RIME) is a recently recognized clinical syndrome characterized by involvement of mucous membranes following a respiratory infection. It typically presents with acute-onset, painful mucosal erosions that primarily affect the oral, urogenital, and ocular membranes, with minimal skin involvement [3].

Our patient’s clinical progression and favorable response to supportive treatment supported the diagnosis of RIME. Diagnosing mucocutaneous eruptions in children requires a structured assessment that encompasses a thorough medication history, appropriate virologic and microbiologic investigations, and, when indicated, specialist input such as dermatology consultation. These steps help distinguish infectious etiologies from drug-related or autoimmune conditions. In this case, the absence of recent medication use supported an infectious cause.

The proposed diagnostic criteria for RIME include evidence of an active infection accompanied by mucosal involvement. While *Mycoplasma pneumoniae *is the most commonly identified pathogen, other respiratory viruses such as rhinoviruses, enteroviruses, and coronaviruses have also been implicated [1, 7].

Distinguishing RIME from Stevens-Johnson Syndrome (SJS) and erythema multiforme (EM) is often difficult. RIME is associated with more severe mucositis and limited skin involvement, whereas SJS typically involves widespread epidermal detachment and is strongly associated with drug exposure [8]. In our patient, the detection of *Mycoplasma pneumoniae *IgM and enterovirus supported previously reported associations with RIME [6].

This case featured an uncommon finding of urethral involvement. Urethral manifestations, though rare, have been described in the literature [9, 10], as mucosal inflammation in RIME can affect any mucous membrane.

RIME is known for its potential to recur, and children may experience multiple episodes triggered by different pathogens [3, 6]. The underlying pathophysiology remains unclear, but several mechanisms have been proposed, including B-cell proliferation, immune complex deposition, complement activation, and molecular mimicry between keratinocyte antigens and *Mycoplasma *P1 adhesion molecules in genetically predisposed patients. A genetic susceptibility, particularly in individuals carrying HLA-B27 and HLA-B51, has also been hypothesized [11].

Treatment of RIME is primarily supportive. The key components include pain control, mucosal protection, and adequate hydration. Antimicrobial therapy should be targeted to confirmed pathogens. Corticosteroids are frequently used in severe cases; however, their efficacy remains debated due to unproven benefits and potential adverse effects [7]. In our case, the patient improved with antimicrobial therapy and supportive measures alone, without the need for corticosteroid treatment.

An accurate diagnosis of RIME is important, as it can help prevent the unnecessary discontinuation of medications and reduce the risk of misdiagnosing drug hypersensitivity. It also allows for appropriate counseling regarding prognosis and the potential for recurrence [1]. There is emerging evidence that certain human leukocyte antigen (HLA) subtypes may predispose individuals to mucocutaneous hypersensitivity reactions, similar to those observed in Stevens-Johnson syndrome. In particular, associations with HLA-B27 and HLA-B51 have been hypothesized as potential contributors to altered immune responses and susceptibility in RIME [11]. However, these findings remain preliminary, and the role of genetic predisposition in RIME is still under active investigation. Further research is needed to clarify these associations and determine their relevance for personalized management approaches.

## Conclusions

This case highlights the importance of distinguishing RIME from Stevens-Johnson Syndrome (SJS) in children with post-infectious mucocutaneous eruptions. It illustrates uncommon but clinically significant features, including co-infection with *Mycoplasma pneumoniae *and enterovirus, as well as urethral involvement, which expands the recognized spectrum of RIME. Accurate diagnosis is crucial to prevent unnecessary interventions and to inform targeted therapy and supportive care. Educating families about early symptom recognition and ensuring close follow-up during respiratory illness seasons remains important, given RIME’s potential for recurrence. By highlighting these rare findings, our report contributes to a deeper understanding of RIME’s diagnostic challenges and clinical variability, supporting greater awareness among healthcare providers.
